# Supplementary material for: Novel Antioxidant Therapy with the Immediate Precursor to Glutathione, γ-Glutamylcysteine (GGC), Ameliorates LPS-Induced Cellular Stress in In Vitro 3D-Differentiated Airway Model from Primary Cystic Fibrosis Human Bronchial Cells
Source: Antioxidants (Basel). 2020 Nov 30;9(12):1204. doi: 10.3390/antiox9121204 (PMC7760366; doi:10.3390/antiox9121204)
Supplement: Supplementary file 1 [file antioxidants-09-01204-s001.zip › FigS1.pdf]

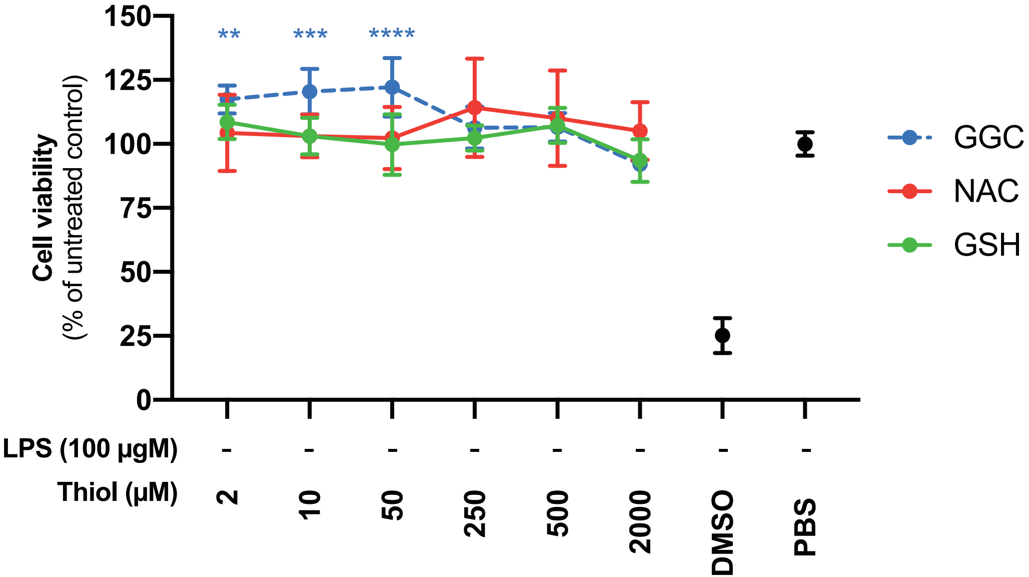

Figure S1. GGC increases metabolic viability in human airway epithelial cells from non-cystic fibrosis donors. Treatment of epithelial cultures with GGC, NAC and GSH for 24 h in a range of concentration (2 to 2000µl). DMSO used as positive cell death control. Metabolic viability measured by MTT assay. Statistical analysis was performed using one-way ANOVA against the PBS control. (* = p<0.05, ** = p<0.01, *** = p<0.001, **** = p<0.0001). Error bars represent SD. Data are from independent experiments performed on non-CF (WT/WT CFTR) airway epithelium cultures from one primary HNEC and one immortalised HBEC, with three technical replicates for each different experimental condition.
