# Supplementary material for: Novel Antioxidant Therapy with the Immediate Precursor to Glutathione, γ-Glutamylcysteine (GGC), Ameliorates LPS-Induced Cellular Stress in In Vitro 3D-Differentiated Airway Model from Primary Cystic Fibrosis Human Bronchial Cells
Source: Antioxidants (Basel). 2020 Nov 30;9(12):1204. doi: 10.3390/antiox9121204 (PMC7760366; doi:10.3390/antiox9121204)
Supplement: Supplementary file 1 [file antioxidants-09-01204-s001.zip › TableS2.docx]

**Table S2. Proteomics analysis statistics.**

| **Comparison (condition 1 vs. condition 2)** | **Number of proteins (cond. 1)** | **Number of proteins (cond. 2)** | **Total Shared Proteins** | **Significantly Changed Proteins** | **Significantly Up Proteins (Cond. 1 relative to Cond. 2)** | **Significantly Down Proteins (Cond. 1 relative to Cond. 2)** |
| --- | --- | --- | --- | --- | --- | --- |
| Mock vs. LPS^+^ | 1858 | 1836 | 1493 | 65 | 44 | 21 |
| LPS^+^ vs. Therapeutic | 1836 | 1908 | 1526 | 56 | 18 | 38 |
| LPS^+^ vs. Prophylactic | 1836 | 1951 | 1561 | 57 | 28 | 29 |
| LPS^+^ vs. T+P | 1836 | 1719 | 1402 | 39 | 15 | 24 |
| Mock vs. GGC | 1858 | 1884 | 1481 | 37 | 26 | 11 |
